# Supplementary material for: A UMLS-based spell checker for natural language processing in vaccine safety
Source: BMC Med Inform Decis Mak. 2007 Feb 12;7:3. doi: 10.1186/1472-6947-7-3 (PMC1805499; doi:10.1186/1472-6947-7-3)
Supplement: Additional file 13 — RAP application directory. Sets up directory for RAP (RDF application for PHP) source code [file 1472-6947-7-3-S13.gz › rap/doc/phpdoc/__filesource/fsource_syntax__syntaxNTripleSerializer.php.html]

xml version="1.0" encoding="iso-8859-1"?


File Source for NTripleSerializer.php


# Source for file NTripleSerializer.php

Documentation is available at NTripleSerializer.php

```
1. <?php

3. // ----------------------------------------------------------------------------------
4. // Class: NTripleSerializer
5. // ----------------------------------------------------------------------------------


11. /**
12. * PHP N-Triple Serializer
13. *
14. * This class serialises models to N-Triple Syntax.
15. *
16. *
17. * <b>History:</b>
18. * <ul>
19. * <li>11-15-2003 Initial version</li>
20. * </ul>
21. *
22. *
23. * @author Daniel Westphal <mail@d-westphal.de>
24. * @version V0.9.1
25. * @package syntax
26. * @access public
27. ***/


31. class NTripleSerializer extends Object {

33. var $debug;
34. var $model;
35. var $res;


41. /**
42. * Constructor
43. *
44. * @access   public
45. */
46. function NTripleSerializer() {
47. $this->debug=FALSE;
48. }

50. /**
51. * Serializes a model to N Triple syntax.
52. *
53. * @param     object Model $model
54. * @return    string
55. * @access    public
56. */
57. function & serialize(&$m) {

59. if (is_a($m, 'DbModel')) $m = $m->getMemModel();

61. $this->reset();
62. if (!HIDE_ADVERTISE) {
63. $this->res .= '# Generated by NTripleSerializer.php from RDF RAP.' .
64. LINEFEED . '# http://www.wiwiss.fu-berlin.de/suhl/bizer/rdfapi/index.html'.
65. LINEFEED . LINEFEED;
66. }

68. foreach ($m->triples as $t) {

70. $s=$t->getSubject();
71. if (is_a($s, 'Blanknode')) {
72. $subject='_:'.$s->getURI();
73. } else {
74. $subject = '<' . ereg_replace(' ', '', $s->getURI()) . '>';
75. }

77. $p=$t->getPredicate();
78. $predicate='<'.ereg_replace(' ', '', $p->getURI()).'>';

80. $o=$t->getObject();
81. if (is_a($o, 'literal')) {
82. $object='"'.$o->getLabel().'"';
83. if ($o->getLanguage()!='') $object.='@'.$o->getLanguage();
84. if ($o->getDatatype()!='') $object.='^^<'.$o->getDatatype().">";
85. } elseif (is_a($o, 'Blanknode')) {
86. $object='_:'.$o->getURI();
87. } else {$object='<'.ereg_replace(' ', '', $o->getURI()).'>';};

89. $this->res.=$subject.' '.$predicate.' '.$object.' .';
90. $this->res.=LINEFEED.LINEFEED;
91. }

93. return $this->res;
94. }


97. /**
98. * Serializes a model and saves it into a file.
99. * Returns FALSE if the model couldn't be saved to the file.
100. *
101. * @access    public
102. * @param     object MemModel $model
103. * @param     string $filename
104. * @return    boolean
105. * @access    public
106. */
107. function  saveAs(&$model, $filename) {

109. // serialize model
110. $n3 = $this->serialize($model);

112. // write serialized model to file
113. $file_handle = @fopen($filename, 'w');
114. if ($file_handle) {
115. fwrite($file_handle, $n3);
116. fclose($file_handle);
117. return TRUE;
118. }else{
119. return FALSE;
120. };
121. }


124. /* ==================== Private Methods from here ==================== */


127. /**
128. * Readies this object for serializing another model
129. * @access private
130. * @param void
131. * @returns void
132. ***/
133. function reset() {
134. $this->res="";
135. $this->model=NULL;
136. }

138. }


141. ?>
```

Documentation generated on Thu, 7 Jul 2005 13:42:18 +0200 by phpDocumentor 1.3.0RC3
